# Posterior Mediastinal Epithelioid Angiosarcoma Arising in Schwannoma: A Case Report and Review of the Literature

**DOI:** 10.3389/fsurg.2021.666389

**Published:** 2021-05-28

**Authors:** Yingming Xiang, Liping Yan, Xia Lin, Xiangyan Zhang, Fangbiao Zhang, Zhijun Wu

**Affiliations:** Lishui Municipal Central Hospital, Lishui, China

**Keywords:** mediastinum, angiosarcoma, schwannoma, surgery, thoracoscopic

## Abstract

Epithelioid angiosarcoma arising in schwannoma is an extremely rare mesenchymal tumor that accounts for only 1 to 2% of all sarcomas. This type of tumor occurs in all parts of the body, most often in the skin and soft tissues and rarely in the mediastinum. The present study describes the case of an asymptomatic, 58-year-old male who presented with epithelioid angiosarcoma in the posterior mediastinum during a physical examination. Enhanced computed tomography of the chest revealed a 3.5 × 3.1-cm mass in the posterior mediastinum. Thoracoscopic mediastinal mass resection was performed under general anesthesia due to the possibility that the tumor was malignant. Pathological examination revealed the presence of angiosarcoma and schwannoma components. Immunohistochemical staining for cluster of differentiation (CD) 31, CD34, early growth response (EGR), vimentin, Sry-related HMG box (SOX)-10 and S-100 was strongly positive. The patient recovered and was discharged on postoperative day 5. Two months postsurgery, the patient returned for evaluation, and no evidence of tumor recurrence was observed.

## Introduction

Epithelioid angiosarcoma arising in schwannoma has been described in a variety of anatomic locations, including the vagus nerve, sciatic nerve, and the adrenal gland, which was initially described by Trassard (1). The majority of epithelioid angiosarcomas occur in the head and neck and rarely in the mediastinum. To the best of our knowledge, the first case of epithelioid angiosarcoma arising in a long-standing mediastinal schwannoma was described by Demiröz et al. (2). In the early stages of a mediastinal mass, patients often do not exhibit clear clinical symptoms. As the tumor grows, patients present with obstructive symptoms that are attributed to the location and size of the tumor, including pain, dyspnea, dysphagia and chest discomfort. Given the rarity of the condition, the available therapeutic methods are not well-developed. However, according to the published literature, complete resection is considered the most effective and successful treatment (2). Postoperative adjuvant chemoradiotherapy is still controversial. The current study describes a rare case of mediastinal epithelioid angiosarcoma arising in a schwannoma in a 58-year-old man and reviews the previously reported cases.

## Case Presentation

**Figure 1:**
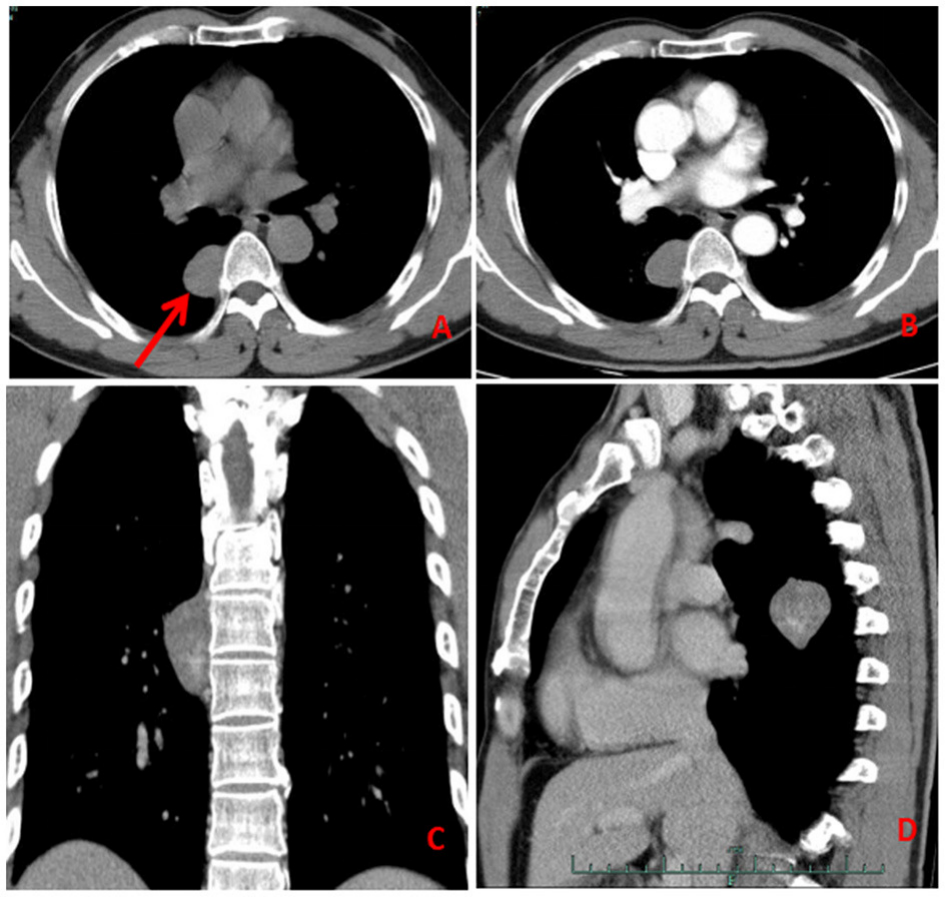
Computed tomography (**A**: axial, **C**: coronal, **D**: sagittal) of the chest showed a mass in the posterior mediastinum (red arrow). The enhancement scan **(B)** was uneven and slightly enhanced.

A 58-year-old, asymptomatic male presented to our hospital (Lishui Municipal Hospital, Lishui, China) for a physical examination. Written informed consent was obtained from the patient for the publication of the present study. The patient had no history of cigarette smoking, hypertensive disease, diabetes mellitus, hepatitis or tuberculosis. Routine blood, liver function, renal function, blood glucose, coagulation function and serum electrolyte test results were all within the normal range. Enhanced computed tomography (CT) (Philips, Brilliance ICT CP 200063) of the chest showed a 3.5 × 3.1-cm mass in the posterior mediastinum, and no other lesions were identified in the thoracic cavity (Figure 1).

**Figure 2:**
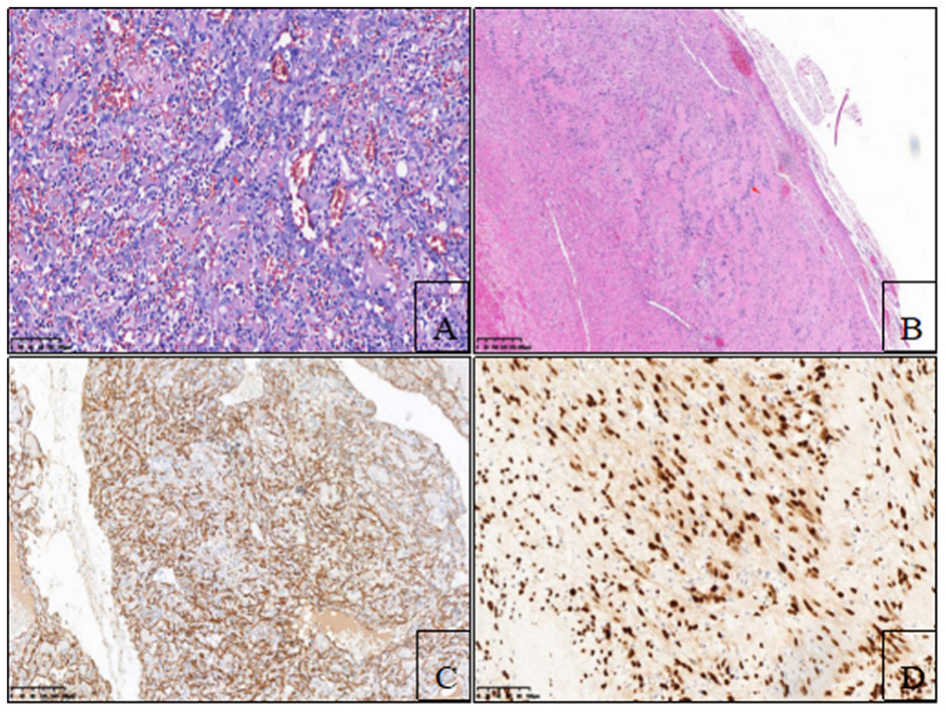
**(A)** Hematoxylin-eosin staining showed that epithelioid angiosarcoma had abundant eosinophilic cytoplasm and vesicular nuclei, with prominent nucleoli (red arrow) (200×). **(B)** Hematoxylin-eosin staining showed that the schwannoma cells were spindle, with abundant cytoplasm and insignificant nucleoli, which were arranged in a palisade shape (red arrow) (40×). **(C)** Epithelioid tumor cells stained positive for CD-31 (100×). **(D)** Spindled schwannian cells were diffusely positive for SOX-10 (200×).

**Figure 3:**
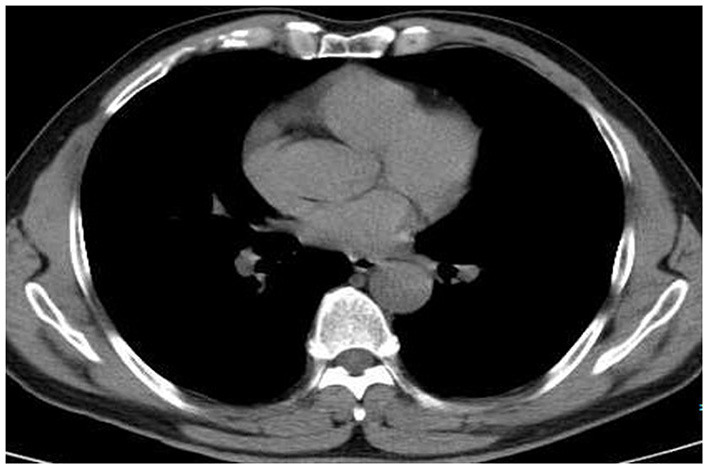
Two months postsurgery, the patient was followed up by chest CT and had no evidence of tumor recurrence.

**Figure 4:**
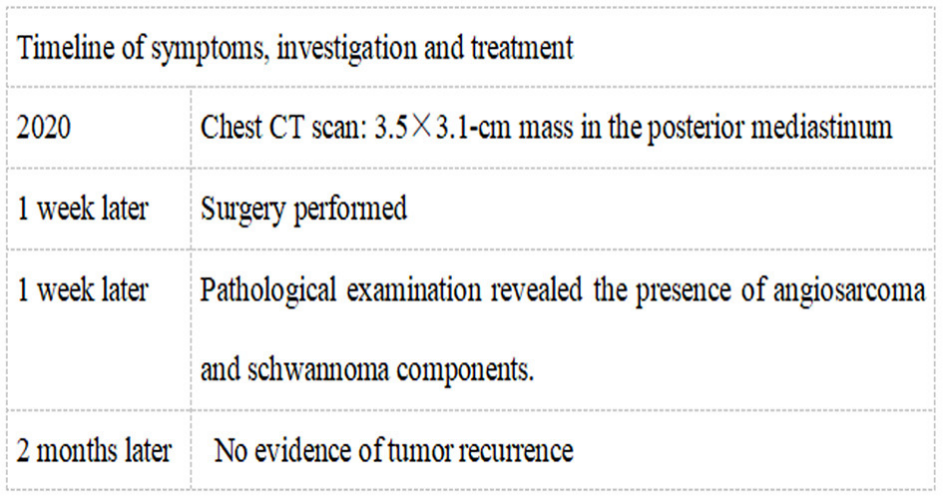
Timeline of symptoms, investigation, and treatment.

A contrast-enhanced CT scan showed an enhanced mass with homogeneous internal density and clear tumor boundaries. A preoperative diagnosis of schwannoma was considered due to the enhanced CT features. Right-side video-assisted thoracoscopic surgery (VATS) was performed under general anesthesia due to the possibility that the mass was malignant. The 4th intercostal incision in the right axillary front was ~3 cm wide and was used as the operating hole; the 7th intercostal incision in the right midaxillary line was ~2 cm wide and was used as the observation hole. Intraoperatively, the tumor had clear boundaries, no invasion of surrounding tissues, and slight toughness. There was no evidence of intraspinal extension of the tumor. The boundary of the tumor was carefully separated with an ultrasonic knife, and the tumor was completely excised. The operation time was 20 min, and the bleeding volume was 20 ml. A chest tube was placed and removed 2 days after the operation. The surgical margins of resection were microscopically negative. Pathological examination revealed the presence of angiosarcoma (Figure 2A) and schwannoma (Figure 2B) components. Immunohistochemical staining for cluster of differentiation (CD) 31 (Figure 2C), CD34, EGR, vimentin, SOX-10 (Figure 2D) and S-100 was strongly positive. The patient rapidly recovered and was eventually discharged. Two months post-surgery, the patient returned for chest CT and had no evidence of tumor metastasis or recurrence (Figure 3 and see timeline in Figure 4).

## Published Case Study Findings

Relevant studies and literature regarding angiosarcoma arising in a schwannoma were searched in databases (PubMed and Web of Science) from January 1980 to January 2020. The text words and MeSH terms “angiosarcoma” and “schwannoma” were used. Tumor characteristics, clinical features, therapeutic strategies and survival outcomes were reviewed, and these data were tabulated. Fifteen articles were identified from database searches, and a total of 21 patients were included (1–15) (Table 1). The patients included 14 males and seven females. The mean age was 53.1 years, ranging from 17 to 74 years. Nine tumors occurred in the neck, one in the mediastinum, one in the buttock, one in the abdomen, one in the inguen, one in the kidney, one in the scalp, one in the chest wall, two in the intracalvarium and three in the thigh. All patients underwent surgery, three patients underwent radiotherapy, three patients underwent chemotherapy, and one patient underwent induction chemotherapy. Nineteen patients received postoperative followup with and a mean length of 15.7 months (range 10 days to 36 months). Seven patients died.

**Table 1:** Cases of angiosarcoma arising in schwannoma.

**References**	**Age (years)**	**Sex**	**Symptoms**	**Size (cm)**	**Localization**	**Treatment**	**Follow-up (mo)**	**Status**
Trassard et al. (1)	65	Male	Intermittent pain	5.5 × 3.5 cm	Right thigh, sciatic nerve	Surgery + CH + RT	36	NED
Demiröz et al. (2)	53	Female	Severe dyspnea and palpitation	18 × 15 × 7 cm	Mediastinum	Surgery	22	NED
Mentzel and Katenkampl (3)	73	Female	Asymptomatic	55 × 45 × 40 mm	Right neck, vagus nerve	Surgery	33	NED
	63	Male	UK	40 × 30 × 20 mm	Right neck, vagus	Surgery	UK	UK
McMenamin and Fletcher (4)	74	Female	Asymptomatic	5.5 × 4.5 × 4.0 cm	Right neck, vagus nerve	CR	33	NED
	40	Female	Painless lump	2.5 cm	Right lower thigh	CR	6	UK
	17	Female	Dyspnea, right-sided chest pain	3 fragments 2–6 cm	Right neck/superior mediastinum, phrenic nerve	ICR + CH + RT	14	Died
	39	Female	Asymptomatic	7.8 × 6.7 × 5.0 cm	Right buttock	ICR	UK	UK
Ito et al. (5)	66	Male	Right-sided facial palsy, headache, gait disturbance and short-term memory loss	UK	Right vestibular nerve	CR	9	Died
Lee et al. (6)	73	Male	Paralysis of the left lower limb	30 cm	Left thigh, sciatic nerve	CR	12	NED
Li et al. (7)	67	Male	Intermittent pain	9 × 6.5 × 8.1 cm	Right abdominal adrenergic nerve	CR	12	NED
	38	Male	UK	7 cm	Right inguinal sciatic nerve	CR + RT	9	Metastasis
	55	Male	Mild pain	5 × 4 × 3.8 cm	Right neck, Vagus nerve	CR	32	NED
Mahajan et al. (8)	41	Male	Swelling in the left submandibular region	11.5 × 6.9 × 6.7 cm	Left neck, vagus nerve	CR + CH	4	Died
Iannaci et al. (9)	56	Male	Lower back pain and hematuria	4 × 2.5 cm	Kidney	Nephrectomy	Few months	Metastasis
He and Li (10)	50	Male	Asymptomatic	5.0 × 3.5 × 3.0 cm	Scalp	Surgery	3	NED
Agarwal et al. (11)	47	Male	Swelling	6 × 6 cm	Left neck, vagus nerve	CR + CH	6	Died
Rückert et al. (12)	50	Male	Pulsatile tender swelling	4.5 × 3.5 × 3.5 cm	Right lateral neck, vagus nerve	CR	15	Metastasis
Shundo et al. (13)	68	Female	Right hemothorax	45 × 45 × 35 mm	Chest wall	Surgery	<1	Died
Ogawa et al. (14)	47	Male	Hoarseness, right ptosis	5 × 6 cm	Right neck, Vagus nerve	Induction CH + Surgery	9	Died
Sakai et al. (15)	33	Male	Right drop foot	UK	Cerebellopontine angle	CR	27	Died

*UK, unknown; NED, no evidence of disease; CH, chemotherapy; CR, complete resection; RT, radiotherapy; ICR, incomplete resection*.

## Discussion

Schwannoma is a benign peripheral nerve sheath tumor that occurs in patients of any age with a slight predilection for adults (9). It is well-known that schwannomas are the most common mediastinal tumors, and malignant transformation is extremely rare. Demiröz et al. first reported mediastinal epithelioid angiosarcoma arising in a schwannoma (2). The case presented here is the second reported case of epithelioid angiosarcoma arising in a mediastinal schwannoma, to the best of our knowledge. The mechanism that leads to schwannoma transformation remains uncertain. Some scholars believe that epithelioid angiosarcoma arises from the vascular structure of the schwannoma. Other scholars believe that chronic vasculostasis, edema and immune response may play an important role in the transformation process (4, 7, 12).

The patients' symptoms are complex and depend on the location and size of the tumor. When the tumor is small, it is often asymptomatic and is detected by physical examination. When the tumor grows, it will compress or invade surrounding tissues, causing pain and difficulty breathing. X-ray and B-ultrasound have no obvious advantage for the early detection of this tumor. CT scans of the chest can detect these tumors early, but the pathology cannot be determined. Whether PET-CT is applicable for such diseases is unclear, and further studies are needed. Benign lesions were considered before surgery in this patient, and PET-CT examination was not performed.

The differential diagnosis of these extremely rare cases includes numerous malignant and benign tumors, including epithelioid malignant peripheral nerve sheath tumors, fibromas, neurofibromas, and hemangiomas. Immunohistochemistry is helpful for differentiating between these tumors. For example, pathological examination revealed the presence of angiosarcoma and schwannoma components. The angiosarcoma cells CD34-, CD31- and EGR-positive, and the schwannoma cells were SOX-10- and S-100-positive.

Surgery is still the preferred treatment for mediastinal tumors, whether they are benign or malignant. Capsular penetration of the tumor or invasion of adjacent tissues are poor prognostic factors because they make complete surgical resection more difficult, if not impossible (4). There is insufficient evidence for postoperative chemoradiotherapy due to the lack of sufficient reported cases. Demiröz et al. (2) described the case of a 53-year-old female who was diagnosed with mediastinal epithelioid angiosarcoma arising in schwannoma. The patient received a posterolateral thoracotomy and radiation oncology for adjuvant therapy. At the 22nd month of follow-up, the patient was asymptomatic (2). Li et al. (7) reported three cases of epithelial angiosarcoma arising from schwannoma that occurred in the left neck, right inguinal region, and right iliac fossa. All patients received surgical treatment, and no antitumor therapy, such as chemoradiotherapy, was performed postoperatively. One of the patients developed pulmonary metastasis 9 months after surgery. Lobectomy combined with mediastinal lymph node dissection was repeated. Chemotherapy was given after the surgery. No tumor recurrence or metastasis was found during the follow-up period of 28 months (7). Similarly, Iannaci et al. (9) reported a case of renal schwannoma transforming into angiosarcoma. Unfortunately, at the time of diagnosis, the patient had already developed inoperable pulmonary metastasis and did not receive antitumor therapy. The patient developed a hemothorax in a short period of time and had a poor prognosis. As the incision margin was negative in the present case, postoperative radiotherapy and chemotherapy were not performed.

## Conclusion

In conclusion, the present study described a rare case of epithelioid angiosarcoma arising in a schwannoma in a male patient. This study revealed four important findings. First, epithelioid angiosarcoma arising in a schwannoma can occur in people of all ages and with no sex predominance. The male-to-female ratio was 1.0:0.5. Second, complete surgical resection presents a safe and effective treatment. Third, preoperative and postoperative chemoradiotherapies are still controversial. Fourth, to the possibility of malignant transformation during the long-term follow-up of patients with schwannoma.

## Data Availability Statement

The original contributions presented in the study are included in the article/supplementary material, further inquiries can be directed to the corresponding author/s.

## Author Contributions

YX and LY drafted the article. XL, FZ, and XZ performed the surgery. ZW made critical revisions for important intellectual content. All authors read and approved the final article.

## Conflict of Interest

The authors declare that the research was conducted in the absence of any commercial or financial relationships that could be construed as a potential conflict of interest.
